# CuFusion: Accurate Real-Time Camera Tracking and Volumetric Scene Reconstruction with a Cuboid

**DOI:** 10.3390/s17102260

**Published:** 2017-10-01

**Authors:** Chen Zhang, Yu Hu

**Affiliations:** College of Computer Science and Technology, Zhejiang University, Hangzhou 310027, China; yudeshui@zju.edu.cn

**Keywords:** real-time reconstruction, SLAM, Kinect sensors, depth cameras, open source

## Abstract

Given a stream of depth images with a known cuboid reference object present in the scene, we propose a novel approach for accurate camera tracking and volumetric surface reconstruction in real-time. Our contribution in this paper is threefold: (a) utilizing a priori knowledge of the precisely manufactured cuboid reference object, we keep drift-free camera tracking without explicit global optimization; (b) we improve the fineness of the volumetric surface representation by proposing a prediction-corrected data fusion strategy rather than a simple moving average, which enables accurate reconstruction of high-frequency details such as the sharp edges of objects and geometries of high curvature; (c) we introduce a benchmark dataset CU3D that contains both synthetic and real-world scanning sequences with ground-truth camera trajectories and surface models for the quantitative evaluation of 3D reconstruction algorithms. We test our algorithm on our dataset and demonstrate its accuracy compared with other state-of-the-art algorithms. We release both our dataset and code as open-source (https://github.com/zhangxaochen/CuFusion) for other researchers to reproduce and verify our results.

## 1. Introduction

Real-time camera tracking and simultaneous dense scene reconstruction has been one of the most actively studied problems in computer vision over recent years. The advent of depth cameras based either on structured light (e.g., Asus Xtion, Kinect 1.0) or time-of-flight (ToF) (e.g., Kinect 2.0) sensing offers dense depth measurements directly in real-time as video streams. Such dense depth sensing technologies have drastically simplified the process of dense 3D modeling, which turns the widely available Kinect-style depth cameras into consumer-grade 3D scanners.

KinectFusion [1] is one of the most famous systems for registering each incoming frame of depth images captured during the scanning into one integrated volumetric representation of the scene. An iterative closest point (ICP) algorithm [2] is performed to align the current depth map to the reconstructed volumetric truncated signed distance function (TSDF) [3] surface model to get the camera pose estimation. Each depth measurement is fused into the TSDF model directly to update the reconstruction. A triangulated 3D mesh model could finally be extracted using a Marching Cubes type algorithm [4].

**Figure 1:**
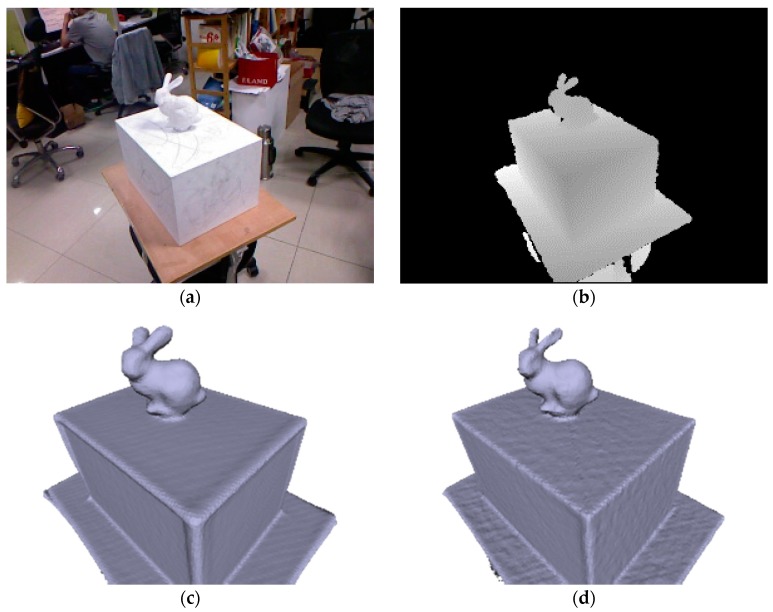
(**a**) Color (not used) and (**b**) depth image from our input sequence “lambunny”; (**c**) KinectFusion: mild accumulated camera drift and simple moving average truncated signed distance function (TSDF) fusion result in reconstruction inflation; (**d**) Our approach, CuFusion, keeps drift free camera tracking with additional constraints of a cuboid reference object and preserves the fidelity of the reconstructed objectives using our prediction-corrected TSDF fusion strategy. Note the sharpness of the cuboid edges and the thinness of the character’s ears of our reconstruction.

Existing geometric alignment approaches based on ICP and its variants [5] are prone to drift in the presence of structure-less surfaces. Drift might be accumulated and even cause the failure of camera tracking when scanning larger man-made environments. Meanwhile, the weighted moving average TSDF fusion strategy makes the assumption of a Gaussian noise model on the depth measurements with a naïve surface visibility predicate that every surface point is visible from all sensor viewpoints [6]. This predicate is only locally true and usually violated due to surface occlusions [1] when scanning around the scene. Although truncation of the signed distance function (SDF) is performed to avoid surfaces interfering, surface blurring and the inflating problem (as shown in Figure 1c) may happen when scanning around tiny objects or sharp geometries in the scene.

Existing algorithms have been proposed to keep globally consistent camera trajectory estimation. Pose graphs are created and optimized when large loop closures are found [7], which may substantially reduce the odometry error accumulation. On the task of scanning small-sized scenes or objects, however, even small camera drift may cause deformation of the reconstruction. We propose a novel algorithm called CuFusion, which particularly focuses on the application of reconstructing small-sized scenes and objects precisely in real-time, with the accuracy of both camera tracking and data fusion significantly improved. With a priori knowledge of the planar faces and occluding contours of the cuboid reference object partly or totally present in the scene, each data frame is aligned against both the reconstructed scene and the localized cuboid model, and thus drift-free camera trajectories are maintained.

The predicate that every surface point is visible from all sensor viewpoints is only locally true due to surface occlusions [1]. In our work, we drop such assumptions and implement a “prediction-corrected” data fusion algorithm to integrate all incoming data into one geometrically consistent 3D model in the global reference frame. Instead of a simple moving average surface reconstruction, our work extends the TSDF representation by adding components storing the locally consistent TSDF value, the pixel ray and surface normal vector in each voxel grid for the detection of the camera view variation and correction of the global TSDF value. Experimental results (Figure 1) show the ability of our fusion method to keep the structural details of surfaces, which is on par with, or better than, existing state-of-the-art reconstruction systems that focus mostly on camera tracking accuracy.

Many scanning and reconstruction systems use both RGB and depth images. Feature-based registration is combined with dense ICP shape matching to estimate the best alignment between consecutive frames. Our system exploits only depth information as input to maximize tracking accuracy for the following reasons: First, some depth cameras such as ASUS Xtion PRO are not accompanied by RGB cameras. Second, for RGB-D cameras which provide both color and depth streams, the spatiotemporal alignment of RGB and depth information in pixel level may not be perfect. Third, by using only depth data, our system enables scanning in complete darkness regardless of the ambient lighting conditions.

We evaluate our algorithm qualitatively and quantitatively using both noiseless synthetic and noisy real-world data captured by a hand-held Kinect. The synthesized data provide both ground-truth (GT) camera trajectories and GT mesh models enabling both the trajectories and reconstructions to be quantitatively evaluated. For real-world image sequences, unfortunately we do not have GT camera trajectories. We 3D printed several rigid models using a high precision 3D printer (http://www.dowell3d.com/3d/3.html) for scanning and evaluate the quality of our reconstructions directly compared with the GT models.

## 2. Related Work

The research into the real-time 3D model reconstruction problem has been extensively studied in recent decades. The advance of range sensing technology has facilitated the development of real-time interactive range scanners for dense 3D surface model acquisition. Such range sensors, particularly on active sensing technologies, could be categorized into different types including laser scanners [8,9], time-of-flight (ToF) [10,11] sensing and structured-light cameras [12]. The introduction of Microsoft’s Kinect—based on structured-light sensing—has brought dense depth sensors to wide consumer-grade accessibility.

KinectFusion [1] of Newcombe et al. is one of the founding systems for real-time dense SLAM, taking a sequence of depth maps streamed from a Kinect-style sensor as the input to create a globally consistent 3D model of the scene. Despite its enlightenment, this algorithm has limitations in several aspects. First, pure geometric alignment of ICP is prone to drift in the presence structure-less surfaces. Second, the regular volumetric representation is memory consuming, which limits the size of the reconstructed model to medium sized rooms, also with limited resolution. Third, it cannot detect loop closures and therefore lacks the ability to recover from accumulating drift, leading to mesh artifacts.

Researchers have been making efforts to address the problems mentioned above. Henry et al. [13] were the first to combine texture feature matching with Generalized-ICP [14] using RGB-D data to reduce drift result from pure geometric alignment and increase the robustness of visual odometry [15]. Loop closure is detected when the previously seen region is revisited, and a pose graph is optimized to create a globally consistent map in [13], as well as in the work of Endres et al. [7,16], Whelan et al. [17,18] and Kerl et al. [19]. Whelan et al. [20] further proposed ElasticFusion, a novel algorithm for loop closure optimization without a pose graph. Moreover, higher-level primitives such as edges [21,22], occluding contours [23], curvature information [24], lines [25] and planes [26,27,28,29] are used as additional information to constrain the pose estimation process.

On dense scene representation, Whelan et al. [17] extended the KinectFusion algorithm spatially to support large unbounded scenes, with a cyclical buffer data structure. Endres et al. [7,16] used an octree-based mapping framework OctoMap [30] to generate a volumetric 3D map of the environment at scale, yet no mesh model is created. Other researchers have been using points and surfels [20,24,31,32,33,34] to represent the scene and render it with the surface-splatting technique [35]. Such point-based scene representation has significantly reduced computational complexity and lowered the memory overhead compared with the volumetric approaches and is therefore adequate for reconstructing large-scale environments. Note that Lefloch et al. [24] use curvature information as an independent surface attribute for their real-time reconstruction, leading not only to camera drift reduction but also to improved scene reconstruction.

However, despite the efforts exerted, both the camera pose estimation and the reconstructed models are far from perfect. On small-sized scenes particularly, slight camera drift may lead to reconstruction deformation and sharp depth edges or highly concave scenes are problematic for these approaches [36]. We tackle these problems and focus on fidelity preservation in this paper.

## 3. Method

**Figure 2:**
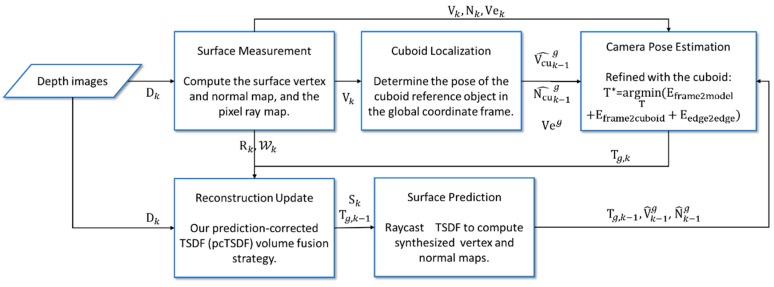
System overview.

We base our work on an open-sourced implementation of the KinectFusion algorithm from the PCL library [37]. Our reconstruction pipeline is illustrated in Figure 2, which is described in detail in the following sections.

### 3.1. Notation

We define the image domain as $\Omega \subset {\mathbb{N}}^{2}$ , and a depth image $D_{k}\;:\Omega \to \mathbb{R}$ at time *k*. We represent the camera pose at time *k* in the global coordinate frame ${ℱ}_{g}$ by a rigid transformation matrix:

(1) $$T_{g,\;k}=\left[\begin{array}{cc} R_{g,\;k} & t_{g,\;k}\\ 0^{T} & 1 \end{array}\right]\in SE\left(3\right),$$

with a 3×3 rotation matrix $R_{g,\;k}\in SO\left(3\right)$ and a 3×1 translation vector $t_{g,\;k}\in {\mathbb{R}}^{3}$ , which transforms a point $p_{k}\in {\mathbb{R}}^{3}$ in the camera coordinate frame ${ℱ}_{k}$ to a global point $p_{g}=R_{g,\;k}p_{k}+t_{g,\;k}\in {\mathbb{R}}^{3}$ . We model the depth camera by the simple pinhole model, and use a constant camera intrinsic matrix K to transform points on the sensor plane into image pixels:

(2) $$K=\left[\begin{array}{ccc} f_{x} & 0 & c_{x}\\ 0 & f_{y} & c_{y}\\ 0 & 0 & 1 \end{array}\right],$$

where $\left(f_{x},\;f_{y}\right)$ are the horizontal and vertical focal lengths and $\left(c_{x},\;c_{y}\right)$ is the image coordinate of the principal point.

We define the 3D back-projection of an image pixel u∈Ω as $p=K^{-1}\dot{u}D\left(u\right)$ , where $\dot{u}\;:={\left(u^{T}|1\right)}^{T}$ is the homogeneous form of u . And inversely, we define the perspective projection of point $p={\left(x,\;y,\;z\right)}^{T}$ as u=π(Kp) , where function $\pi \left(p\right)={\left(x/z,\;y/z\right)}^{T}$ performs perspective projection including de-homogenization process.

Prior to registration, an organized vertex map $V_{k}$ is computed by bilateral-filtering and back-projecting the raw depth image $D_{k}$ . The normal map $N_{k}$ is computed using the PCA method. Given the camera pose $T_{g,\;k}$ at time *k*, we could transform both $V_{k},{\;N}_{k}$ to the global frame of coordinate:

(3) $$\{\begin{array}{l} {\dot{V}}_{k}^{g}\left(u\right)=T_{g,\;k}{\dot{V}}_{k}\left(u\right)\\ N_{k}^{g}\left(u\right)=R_{g,\;k}N_{k}\left(u\right) \end{array},$$

### 3.2. Cuboid Localization

Given a depth image $D_{k}$ and a rectangular cuboid with edge lengths ${𝒫}_{cu}=(a,\;b,\;c)$ present in the image, we localize the cuboid and calculate its pose in the global coordinate frame ${ℱ}_{g}$ . Live depth frames will be latterly aligned against the reference cuboid when scanning around it to mitigate the accumulating camera drift.

**Figure 3:**
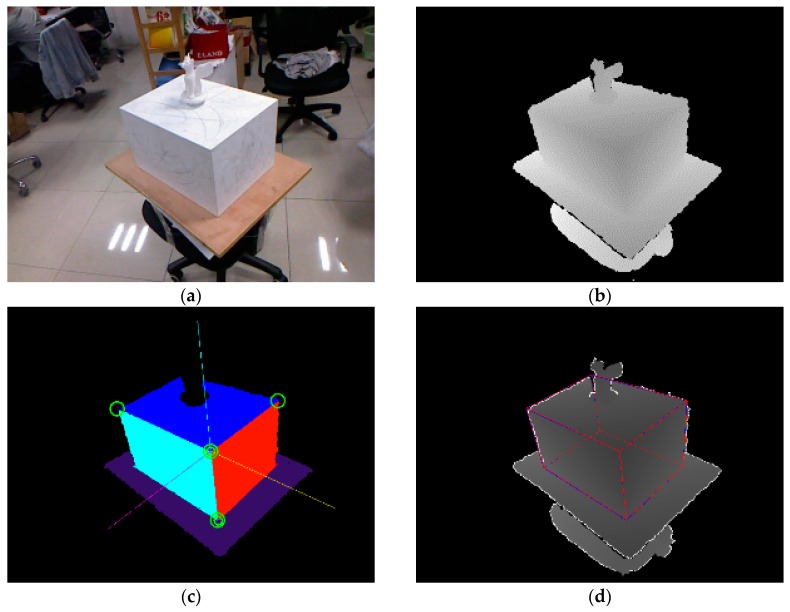
(**a**) Color (not used) and (**b**) depth image from our input sequence “wingedcat;” pixels with depth values larger than 1.5 m are truncated in the depth image; (**c**) Segmented planes obtained by the AHC algorithm [38] are labeled with random colors, the cuboid is localized with its vertices marked as green circles, and the axes of the cuboid frame are drawn in CMY colors; (**d**) The localized cuboid is drawn as a red wireframe in the depth image, and the “contour generators” proposed in [23] are drawn as white lines.

We first perform plane segmentation using the Agglomerative Hierarchical Clustering (AHC) algorithm [38], as illustrated in Figure 3c. Then we check the orthogonality of the segmented planes. Two planes are considered to be orthogonal if the angle $\Theta_{p}$ between their normal vectors is approximately 90° (i.e., $|\Theta_{p}-90{}^{\circ}|<\varepsilon_{\Theta };\;\varepsilon_{\Theta }=5{}^{\circ}).$ Once we find three planes that are orthogonal to each other, we check the length of the intersecting line segments between the planes. If the three line segments’ lengths match the cuboid edge length parameter ${𝒫}_{cu}$ approximately (differences below a threshold $\varepsilon_{𝒫}=10\;\mathrm{mm}$ ), we claim to find the cuboid and mark the three planes as its adjacent planes.

We consequently define the cuboid coordinate frame of reference. We set frame origin $O_{cu}$ to the intersection point of the three orthogonal planes, and draw the system axes from the normal vectors. Due to the inaccuracy of the depth measurement and camera intrinsic calibration, orthogonality between the normal vectors of the segmented adjacent planes are not guaranteed strictly. We obtain the nearest orthogonal axes $\left[X_{cu},\;Y_{cu},\;Z_{cu}\right]$ of the frame by solving the Orthogonal Procrustes Problem. The cuboid pose in the camera frame at time *k* is:

(4) $$T_{k,\;cu}=\left[\begin{array}{cc} R_{k,\;cu} & t_{k,\;cu}\\ 0^{T} & 1 \end{array}\right]\in SE\left(3\right),$$

(5) $$R_{k,\;cu}=\left[X_{cu},\;Y_{cu},\;Z_{cu}\right],$$

(6) $$t_{k,\;cu}=O_{cu}^{T},$$

**Figure 4:**
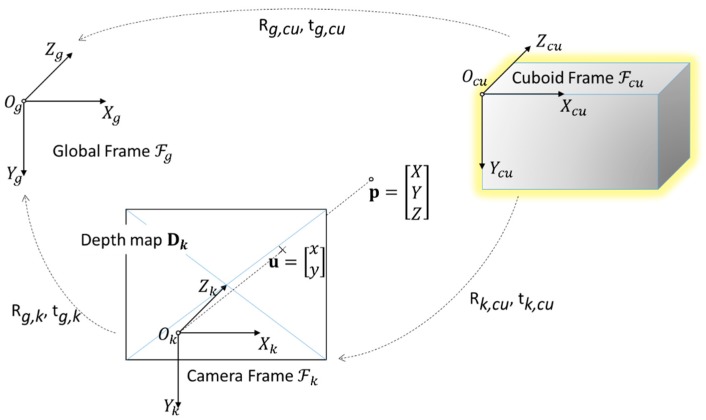
Illustration of the notations used in this paper.

Assuming the camera pose $T_{g,\;k}$ at time *k* is known, the cuboid pose $T_{g,\;cu}=\left[\begin{array}{cc} R_{g,\;cu} & t_{g,\;cu}\\ 0^{T} & 1 \end{array}\right]$ in the global frame of coordinate could then be derived: $T_{g,\;cu}=T_{g,\;k}{\;T}_{k,\;cu}$ . Figure 4 illustrates the notations used in the paper.

### 3.3. Camera Pose Estimation

Since we use depth maps as input sequences, only geometric alignment is performed. For each input frame $D_{k}$ at time *k*, we estimate the pose $T_{g,\;k}$ of the depth camera frame ${ℱ}_{k}$ with respect to the global frame ${ℱ}_{g}$ by registering the live depth map to both the global reconstructed surface model and the cuboid reference object.

A. Frame to Model Registration

Given the implicit TSDF surface model S , the surface prediction w.r.t. the camera pose $T_{g,\;k-1}$ is obtained as an organized vertex and normal map $\left({\hat{V}}_{k-1},\;{\hat{N}}_{k-1}\right)$ , and transformed into the global frame as $\left({\hat{V}}_{k-1}^{g},\;{\hat{N}}_{k-1}^{g}\right)$ . For frame-to-model registration, a transformation $T_{g,\;k}$ is pursued to minimize the point-to-plane error between $T_{g,\;k}V_{k}$ and ${\hat{V}}_{k-1}^{g}$ :

(7) $$E_{frame2model}\left(T_{g,\;k}\right)=\sum_{\left(u,\;\hat{u}\right)\in K_{1}}{\left(\left(T_{g,\;k}{\dot{V}}_{k}\left(u\right)-{\hat{V}}_{k-1}^{g}\left(\hat{u}\right)\right){\hat{N}}_{k-1}^{g}\left(\hat{u}\right)\right)}^{2},$$

where $K_{1}=\left\{\left(u,\;\hat{u}\right)\right\}$ is the set of correspondences obtained by projective data association [1]:

(8) $$\hat{u}=\pi \left({K\;\tilde{T}}_{k-1,\;k}{\dot{V}}_{k}\left(u\right)\right),$$

${\tilde{T}}_{k-1,\;k}$ denotes the transformation from current time *k* to time (*k* − 1) during each ICP iteration.

B. Frame to Cuboid Registration

Assuming the cuboid pose w.r.t., the global coordinate frame is already known. For each camera pose $T_{g,\;k}$ , per-pixel ray casting is performed on the global cuboid to synthesize a proxy depth map ${\hat{D}}_{k}^{cu}$ . An organized vertex and normal map in the global frame as $\left({\hat{V_{\mathrm{cu}}}}_{k-1}^{g},\;{\hat{N_{\mathrm{cu}}}}_{k-1}^{g}\right)$ is then obtained using back projection of the depth map and local to global transformation. Similar to the frame-to-model registration, a frame is aligned against the cuboid surface in the global coordinate frame by minimizing the point-to-plane error:

(9) $$E_{frame2cuboid}\left(T_{g,\;k}\right)=\sum_{\left(u,\;\hat{u}\right)\in K_{2}}{\left(\left(T_{g,\;k}{\dot{V}}_{k}\left(u\right)-{\hat{V_{\mathrm{cu}}}}_{k-1}^{g}\left(\hat{u}\right)\right){\hat{N_{\mathrm{cu}}}}_{k-1}^{g}\left(\hat{u}\right)\right)}^{2},$$

In addition, we adopt the edge-to-edge error metric as a constraint to mitigate the potential camera drift. Given the inpainted depth map $D_{k}^{\prime }$ , we find the edge points (i.e., pixels at depth discontinuities) on the live depth map along the contour generator set $C_{k}$ as proposed in [23]:

(10) $$C_{k}=\left\{s\in D_{k}\;:\exists t\in N_{s}^{8},\;s.t.{\;D}_{k}^{\prime }\left(s\right)-D_{k}^{\prime }\left(t\right)>\delta_{c}\right\},$$

where $N_{s}^{8}$ is the 8-neighborhood of pixel $s\in D_{k}$ and $\delta_{c}$ is the depth discontinuity threshold, set to 50 mm according to the sensor noise magnitudes [39]. Figure 3d demonstrates the contour generators with white lines labeled on the depth map. Edge points set ${\mathrm{Ve}}_{k}$ of the live depth map is obtained by back-projection of $C_{k}$ .

On the other hand, the cuboid edges are discretized into a 3D point set ${\mathrm{Ve}}_{g}^{cu}$ in the global frame with an interval of 1 mm. ${\mathrm{Ve}}_{g}^{cu}$ is invariant to the camera pose, and is obtained once the cuboid is successfully localized, prior to the ICP registration procedure. We also set up a KD-tree over ${\mathrm{Ve}}_{g}^{cu}$ beforehand for fast correspondence search for each point in ${\mathrm{Ve}}_{k}$ . The edge-to-edge error to minimize is:

(11) $$E_{edge2edge}\left(T_{g,\;k}\right)=\sum_{\left(s,\;t\right)\in K_{3}}{\left(\left(T_{g,\;k}{\dot{\mathrm{Ve}}}_{k}\left(s\right)-{\mathrm{Ve}}_{g}^{cu}\left(t\right)\right){\hat{N_{\mathrm{cu}}}}_{k-1}^{g}\left(t\right)\right)}^{2},$$

where $K_{3}=\left\{\left(s,\;t\right)\right\}$ is the correspondence set obtained by nearest neighbor search with KD-tree.

C. Joint Optimization

We combine Equations (7), (9) and (11) to form a joint cost function:

(12) $$E_{track}=E_{frame2model}+{𝓌}_{f2c}E_{frame2cuboid}+{𝓌}_{e2e}E_{edge2edge},$$

where ${𝓌}_{f2c}$ and ${𝓌}_{e2e}$ are the weights that determine the influence of correspondences on the cuboid surfaces and edges. When setting ${𝓌}_{f2c}={𝓌}_{e2e}=0$ , our optimization objective is equivalent to KinectFusion. We set ${𝓌}_{f2c}=1$ and ${𝓌}_{e2e}=4$ in our experiments empirically, enforcing the constraint of the edge correspondences.

The camera pose $T_{g,\;k}$ is then obtained by minimizing the overall cost function $E_{track}$ iteratively. A linear approximation is made to solve the system, assuming the orientation change between consecutive frames is very small [1,40]. Using the small angle assumption at each iteration, we approximate the incremental rotation matrix as:

(13) $$R_{inc}=[\begin{array}{ccc} 1 & -\gamma & \beta \\ \gamma & 1 & -\alpha \\ -\beta & \alpha & 1 \end{array}],$$

where α , β , and γ are the rotation in radians about the *X*, *Y*, and *Z* axis, respectively. Similar to KinectFusion, we compute and sum the linear system in parallel on the GPU, and solve it on the CPU using a Cholesky decomposition.

### 3.4. Improved Surface Reconstruction

**Figure 5:**
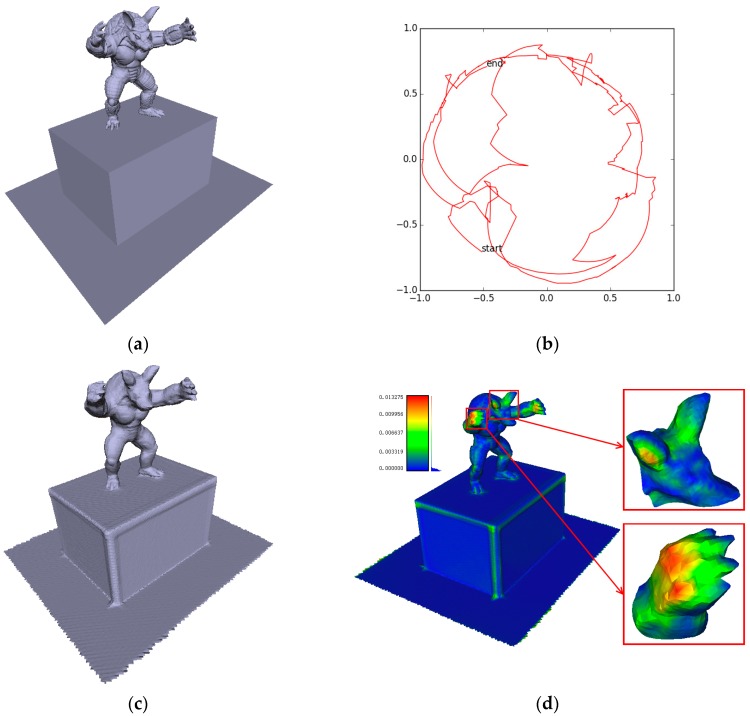
(**a**) Ground-truth (GT) mesh surface model and (**b**) GT camera trajectory which are used for depth image generation; (**c**) Surface reconstruction with GT camera trajectory as input using the simple moving average TSDF fusion strategy; (**d**) A heat map is used to visualize the cloud-to-mesh distances from reconstructed point cloud to the GT mesh. Note the inflation of the cuboid edges, the claws and ears of the armadillo character.

Although we are trying to stabilize camera tracking, surface reconstruction is yet to be perfect. The TSDF volumetric representation allows for online surface extraction as a polygon mesh, while the simple moving average TSDF fusion strategy proposed in KinectFusion suffers from the inflation problem, and lower the reconstruction accuracy. Figure 5 illustrates one of our synthetic datasets “armadillo.” Even with noiseless depth images and GT camera trajectory as input, surface reconstruction is smoothed and inflated, particularly at the cuboid edges, the claws and ears of the armadillo, which is far less satisfactory than the GT surface model.

**Figure 6:**
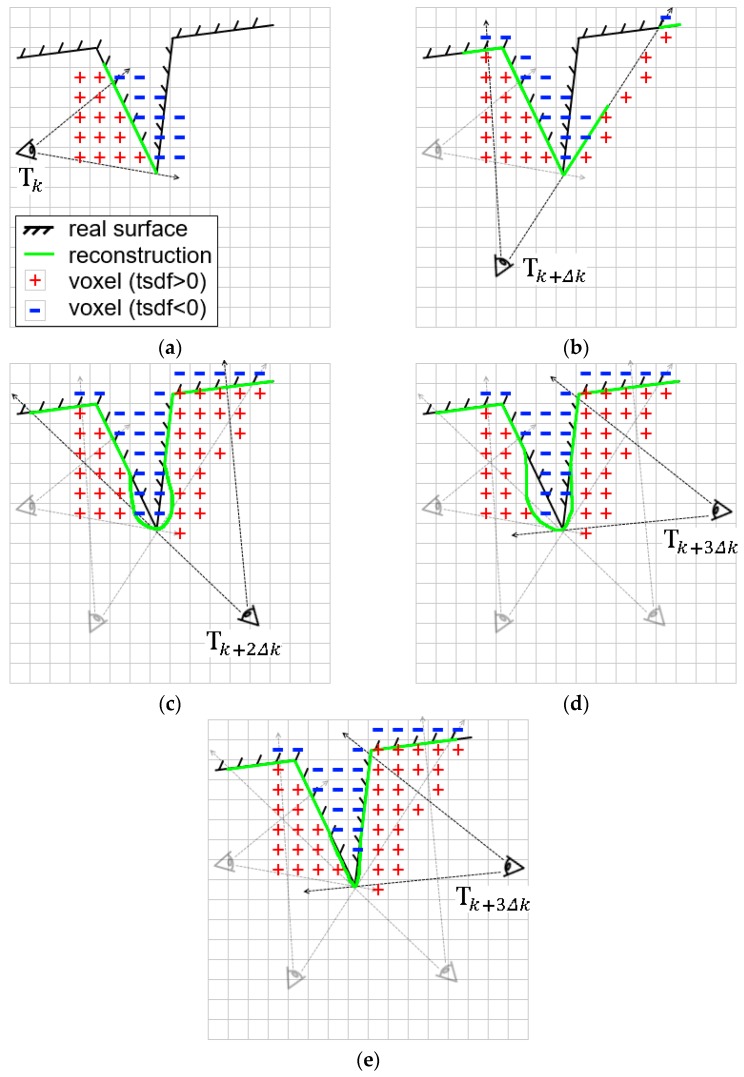
Illustration of the TSDF update process of KinectFusion [1]. (**a**–**d**) denote four different camera poses and update of surface reconstruction (green line) at time k, (k+Δk), (k+2Δk), (k+3Δk) respectively. Note the inflated reconstruction of the highly-convex surface (black line) during the camera movement; (**e**) denotes our reconstruction at time (k+3Δk) . Compared with (**d**), our result preserves the sharpness of the protrusion area of the surface.

**Figure 7:**
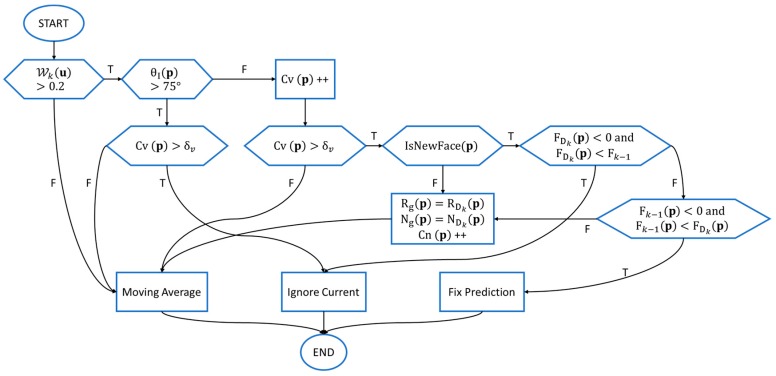
Description of our TSDF fusion algorithm as a flowchart.

The reason for fusion inflation is illustrated in Figure 6. Due to the simple moving average TSDF fusion algorithm based on the predicate that every surface point is visible from all sensor viewpoints [6], voxel grids with negative TSDF values interfere with the positive ones. To tackle this problem, we extend the storage of TSDF S(p) from the truncated signed distance value F(p) and its weight W(p) to:

(14) $$S\left(p\right)\mapsto \left[F\left(p\right),\;W\left(p\right),\;F\prime \left(p\right),\;W\prime \left(p\right),{\;R}_{g}\left(p\right),{\;N}_{g}\left(p\right),\;\mathrm{Cv}\;\left(p\right),\;\mathrm{Cn}\;\left(p\right)\right],$$

where for each voxel grid p :

1.
[F(p), W(p)] are the original TSDF components, and [F′(p), W′(p)] are “ghost” distance value and weight for correction of the existing TSDF prediction;
2.
$R_{g}\left(p\right)$ and $N_{g}\left(p\right)$ are the view ray and the normal vector in the global coordinate frame respectively, which are used to check if a new surface patch is observed from a different view;
3.
Cv (p) and Cn (p) are two integer counters as the confidence indices of voxel p and its normal vector $N_{g}\left(p\right)$ . When $\mathrm{Cv}\;\left(p\right)>\delta_{v}$ , we think the distance value F(p) of voxel has been robustly estimated; when $\mathrm{Cn}\;\left(p\right)>\delta_{n}$ , the normal vector $N_{g}\left(p\right)$ is believed to be stable enough against the measurement noise. A simple Boolean semantic function is defined to check if a new face is observed from a new view point:
(15) $$\mathrm{IsNewFace}\left(p\right)=\mathrm{True}\;\mathrm{iff}\;\{\begin{array}{c} \mathrm{Cn}\;\left(p\right)>\delta_{n},\;\mathrm{and}\\ \mathrm{Angle}\left(R_{g}\left(p\right),\;R_{D_{k}}\left(p\right)\right)>\theta_{r},\;\mathrm{and}\\ \mathrm{Angle}\left(N_{g}\left(p\right),\;N_{D_{k}}\left(p\right)\right)>\theta_{n}. \end{array},$$
  where the thresholds are set to $\delta_{v}=15,{\;\delta }_{n}=5,{\;\theta }_{r}=15{}^{\circ},{\;\theta }_{n}=30{}^{\circ}$ empirically. We define a weight map $W_{k}$ for each input frame $D_{k}$ :
(16) $$W_{k}\left(u\right)=cos\left(\theta_{I}\right)*\frac{L_{k}\left(u\right)}{D_{k}\left(u\right)},$$
  with $\theta_{I}=\mathrm{Angle}\left(R_{D_{k}}\left(p\right),{\;N}_{D_{k}}\left(p\right)\right)$ denoting the incidence angle of the view ray to the surface, and $L_{k}$ is a distance transform map obtained from the contour generator map $C_{k}$ . For each grid p in the TSDF volume, we obtain the adaptive fusion weight $W_{k}\left(p\right)$ and the truncation distance threshold $\mu_{k}\left(p\right)$ :
(17) $$\{\begin{array}{c} W_{D_{k}}\left(p\right)=W_{base}*W_{k}\left(u\right)\\ \mu_{D_{k}}\left(p\right)=\mu_{base}*W_{k}\left(u\right) \end{array},$$
  where u is the projection of p given the camera pose $T_{g,\;k}$ , and $W_{base}$ , $\mu_{base}$ are the base weight and base truncation distance which are set empirically. Our prediction-corrected TSDF fusion algorithm is then detailed as a flowchart in Figure 7. We categorize the fusion procedure into three sub-strategies:
  ***Moving Average*:** Identical to the TSDF update procedure of KinectFusion, simple moving average TSDF fusion is performed when a voxel has high uncertainty (e.g., at glancing incidence angle or too close to the depth discontinuity edge):
(18) $$\{\begin{array}{c} F_{k}\left(p\right)=W_{k-1}\left(p\right)F_{k-1}\left(p\right)+W_{D_{k}}\left(p\right)F_{D_{k}}\left(p\right)\\ W_{k}\left(p\right)=W_{k-1}\left(p\right)+W_{D_{k}}\left(p\right) \end{array},$$
  ***Ignore Current*:** We ignore the TSDF value at the current time when a previously robustly estimated voxel is at glancing incidence angle along the view ray. This is also the case when the current TSDF value with higher uncertainty is observed from a new perspective.
  ***Fix Prediction*:** When a voxel with previously stable TSDF value $F_{k-1}\left(p\right)<0$ is observed to increase from a new point of view—either with $F_{D_{k}}\left(p\right)>0$ or $\left(F_{D_{k}}\left(p\right)\langle 0{\;\mathrm{and}\;F}_{D_{k}}\left(p\right)\rangle F_{k-1}\left(p\right)\right)$ —we believe the live TSDF estimation is more trustworthy as a correction of the previous prediction. In the case of measurement noise, we fuse the live estimation into the ghost storage:
(19) $$\{\begin{array}{c} F_{k}^{\prime }\left(p\right)=W_{k-1}^{\prime }\left(p\right)F_{k-1}^{\prime }\left(p\right)+W_{D_{k}}\left(p\right)F_{D_{k}}\left(p\right)\\ W_{k}^{\prime }\left(p\right)=W_{k-1}^{\prime }\left(p\right)+W_{D_{k}}\left(p\right) \end{array},$$
  and replace the global TSDF with the ghost storage when $W_{k}^{\prime }\left(p\right)$ is above a threshold:
(20) $$\{\begin{array}{c} F_{k}\left(p\right)=F_{k}^{\prime }\left(p\right)\\ W_{k}\left(p\right)=W_{k}^{\prime }\left(p\right) \end{array},$$

Note the update of $\left[R_{g}\left(p\right),{\;N}_{g}\left(p\right),\;\mathrm{Cv}\left(p\right),\;\mathrm{Cn}\left(p\right)\right]$ is performed independently from the three fusion strategies. With our subdivided fusion algorithm, different surface areas are reconstructed elaborately, resulting in the good preservation of high-curvature surface areas, as illustrated in Figure 6e.

## 4. Evaluation

We compare our algorithm with four other dense tracking and mapping approaches: KinectFusion [1] (PCL implementation [37]), the work of Zhou et al. [23], ElasticFusion [20] of Whelan et al., and the NICP algorithm [32] of Serafin et al. ElasticFusion jointly aligns RGB and depth information, while the other four are pure depth camera tracking and reconstruction approaches. We set the weight $w_{rgb}=0$ for RGB alignment component in ElasticFusion, to make it relies only on depth camera tracking as in others’ work. On the evaluation of NICP, we run their CPU implementation offline at full resolution, with default configuration (only the camera parameters are updated). We use the point clouds for reconstruction accuracy evaluation.

Since the scales of our scanned objectives are small, we use a volume of size $1{\;m}^{3}$ with $256^{3}$ voxels for all the compared algorithms, where each voxel is approximately $3.9{\;\mathrm{mm}}^{3}$ .

### 4.1. Dataset

A. Noiseless Synthetic Data

**Figure 8:**
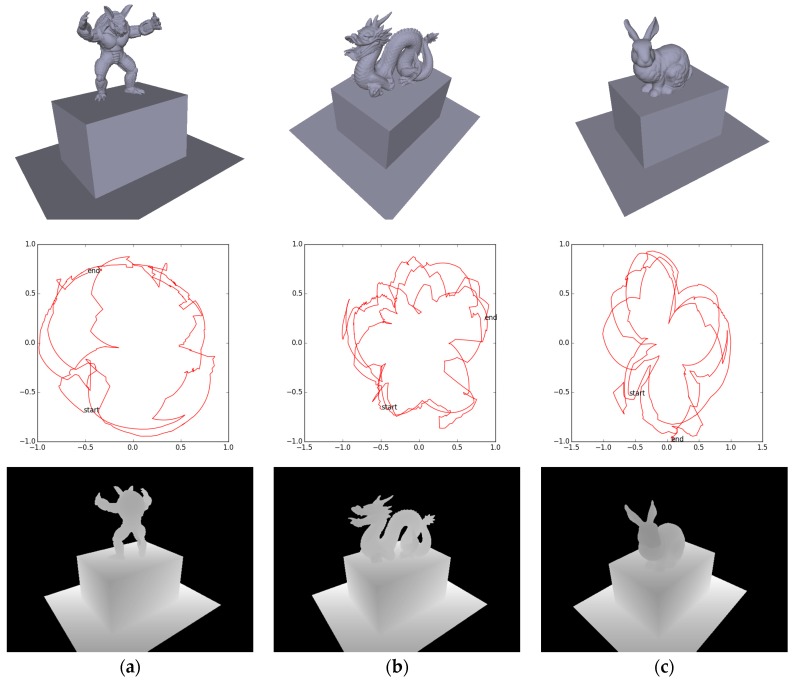
Synthetic data. The (**a**) armadillo; (**b**) dragon; and (**c**) bunny are set upright on top of a cuboid ( $400\times 300\times 250{\;\mathrm{mm}}^{3}$ ) respectively. The top row shows the snapshot of the GT models, the middle row shows the GT camera trajectories (top view), and the bottom row shows the generated depth images (at time 0).

We synthesize three depth image sequences with ground-truth (GT) mesh surface models and GT camera trajectories. A camera intrinsic matrix $K_{s}$ is given to generate images of resolution 640×480 , as shown in Table 1. We choose from “The Stanford Models” [41] the armadillo, dragon and bunny, and scale and place them respectively on top of a synthetic cuboid of edge lengths ${𝒫}_{cu}=(400,\;300,\;250)$ mm. We then move the camera freely around the scene to generate GT trajectories and depth images, as illustrated in Figure 8. Note that neither the depth measurement noise nor the motion blur is modeled and the only measurement inaccuracy comes from data type casting from floats to integers when saving the depth images.

B. Noisy Real-World Data

**Figure 9:**
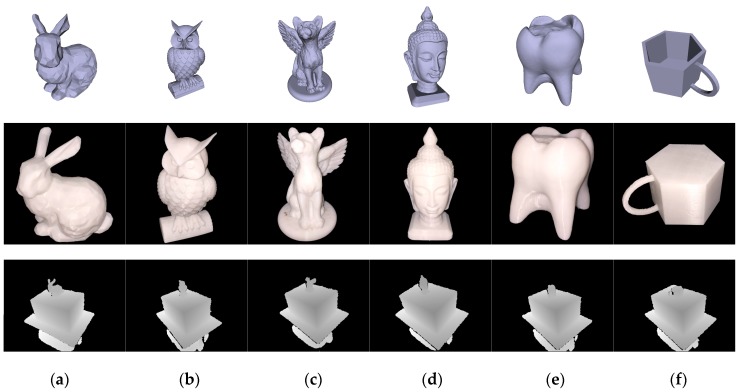
Real-world data: (**a**) lambunny; (**b**) owl; (**c**) wingedcat; (**d**) buddhahead; (**e**) tooth; and (**f**) mug. The top row shows the snapshots of the GT models, the middle row shows the 3D printed rigid objects, and the bottom row shows the depth images of models placed on our man-made cuboid resting on a turntable.

We manufacture six rigid objects using a 3D printer and put them on a precisely manufactured cuboid with dimensions $400\times 300\times 250\;{\mathrm{mm}}^{3}$ , same as the one used in our synthetic data. The cuboid is placed on a turntable which is turned by hand, and we held and moved a Kinect camera slowly to perceive more details of the objectives. 640×480 pre-aligned RGB-D images are generated at 30 Hz , with the camera intrinsic matrix $K_{r}$ (Table 1). We pre-process the depth sequences by truncating depth pixels of values larger than 1.5 m, to remove static background areas. Figure 9 demonstrates our GT mesh models, the 3D printed objects and the captured depth images with the scanning objectives placed on top of the cuboid reference object. Note that in data “lambunny,” a simplified bunny model with merely 640 vertices and 1247 faces is used, and in data “mug,” a regular hexagonal mug resting upside down on the cuboid is scanned.

### 4.2. Error Metrics

On synthetic data, both GT camera trajectories and GT mesh surfaces are provided. We quantify the accuracy of camera trajectory using absolute trajectory error (ATE) proposed by Sturm et al. [42], and evaluate the root mean squared error (RMSE) of the translational components over all time indices, which gives more influence to outliers. We further quantify the surface reconstruction accuracy using the cloud to mesh (C2M) distance metric [43] after aligning the GT model with the reconstructed model using the CloudCompare software [44]. We use two standard statistics: Mean and Std. over the C2M distances for all vertices in the reconstruction. On our real-world data, GT camera trajectories are not available nor do we have GT surface models of the entire scenes. We focus on the evaluation of the reconstructed 3D printed models using the C2M error metric.

### 4.3. Camera Trajectory Accuracy

We evaluate the absolute trajectory error (ATE) of the camera trajectories on synthetic depth image sequences. Although planar surfaces of the cuboid occupy the majority of the depth images, KinectFusion [1], Zhou et al. [23], ElasticFusion [20] and our approach achieve decent camera trajectories without prominently accumulating drift, as listed in Table 2. However, NICP [32] produces inaccurate trajectories with ATE up to hundreds of millimeters. With the additional information from the cuboid a priori, our approach significantly outperforms the reference algorithms, reducing the RMS odometry error from 3~8 mm to less than 2 mm.

**Figure 10:**
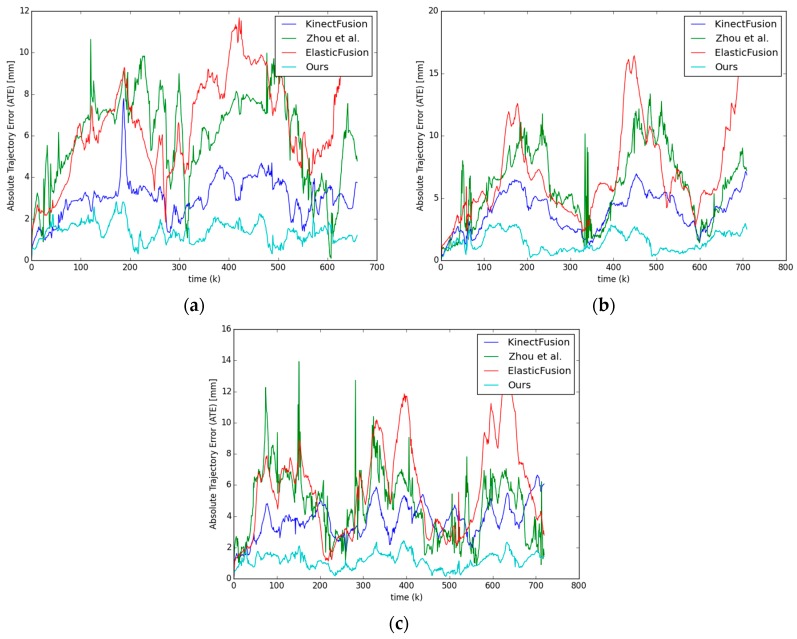
Illustration of per frame ATE on the synthetic (**a**) armadillo; (**b**) dragon; and (**c**) bunny data sequences.

Since the errors of all the trajectory estimations (NICP excluded) on synthetic data are small ( <10 mm), we plot the per frame ATE (as in Figure 10) for each algorithm (NICP excluded) rather than the trajectory overviews. Our approach (cyan line) keeps the least drift on most of the frames compared with the other three algorithms.

### 4.4. Surface Reconstruction Accuracy

**Figure 11:**
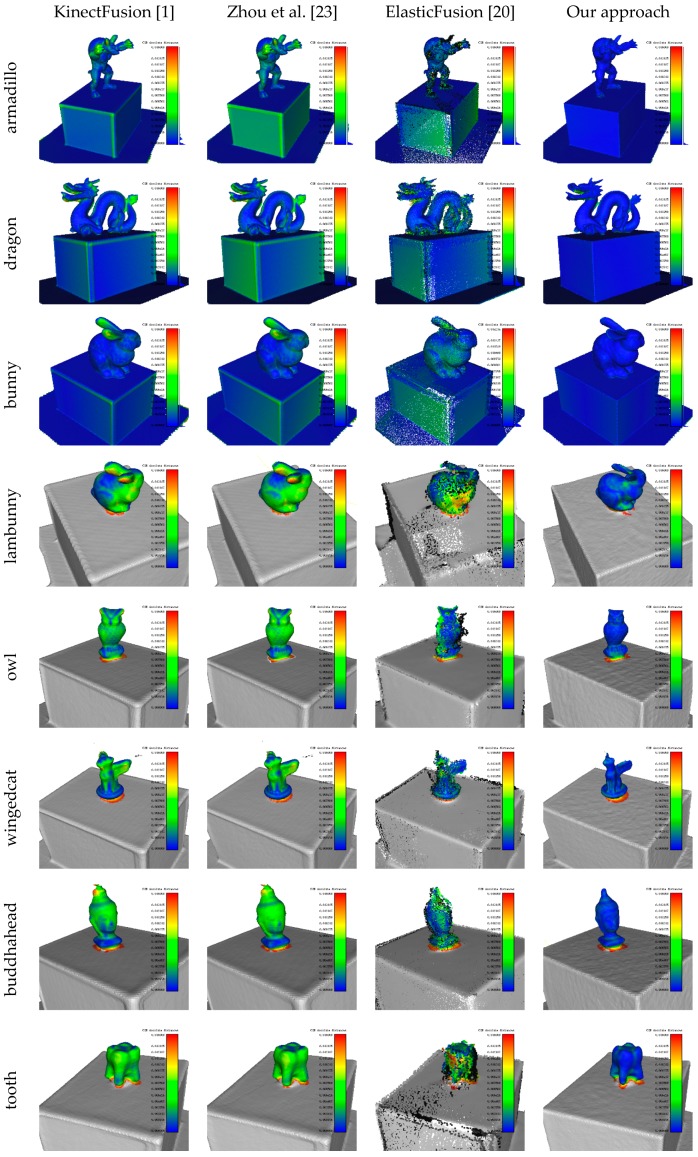

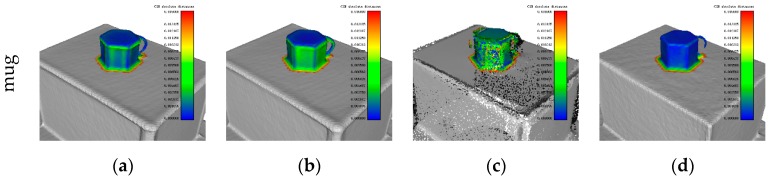
Heat maps of cloud to mesh (C2M) distances for qualitative evaluation of the reconstructions. The compared algorithms are (**a**) KinectFusion [1]; (**b**) Zhou et al. [23]; (**c**) ElasticFusion [20]; and (**d**) our approach. Rows 1~3 are reconstructions of the synthetic data, rows 4~9 are the real-world reconstructions (only the 3D printed objectives are evaluated, with other areas of the scenes grayed out). The scale of the color bar is 0~15mm among all the tests.

The surface reconstruction accuracy is evaluated with the cloud to mesh (C2M) distances between the reconstructions and the ground-truth mesh models. For our synthetic data, GT models of the whole scenes are provided while for our real-world data, we have only GT models for the 3D printed objectives placed on the reference cuboid. Surface reconstructions are first aligned against the GT models for C2M distance computation, and heat maps of the C2M distances are plotted in Figure 11 for qualitatively reconstruction accuracy evaluation. Rows 1~3 show the reconstruction of the synthetic data inputs, and rows 4~9 show the real-world ones. The outputs of ElasticFusion in column 3 are not watertight, since it outputs clouds instead of meshes. NICP is excluded from comparison, since its inaccurate camera trajectories result in invalid scene clouds on our benchmark dataset. Note how tightly our approach preserves the scale of the reconstruction and maintains high-fidelity particularly on sharp geometries.

**Figure 12:**
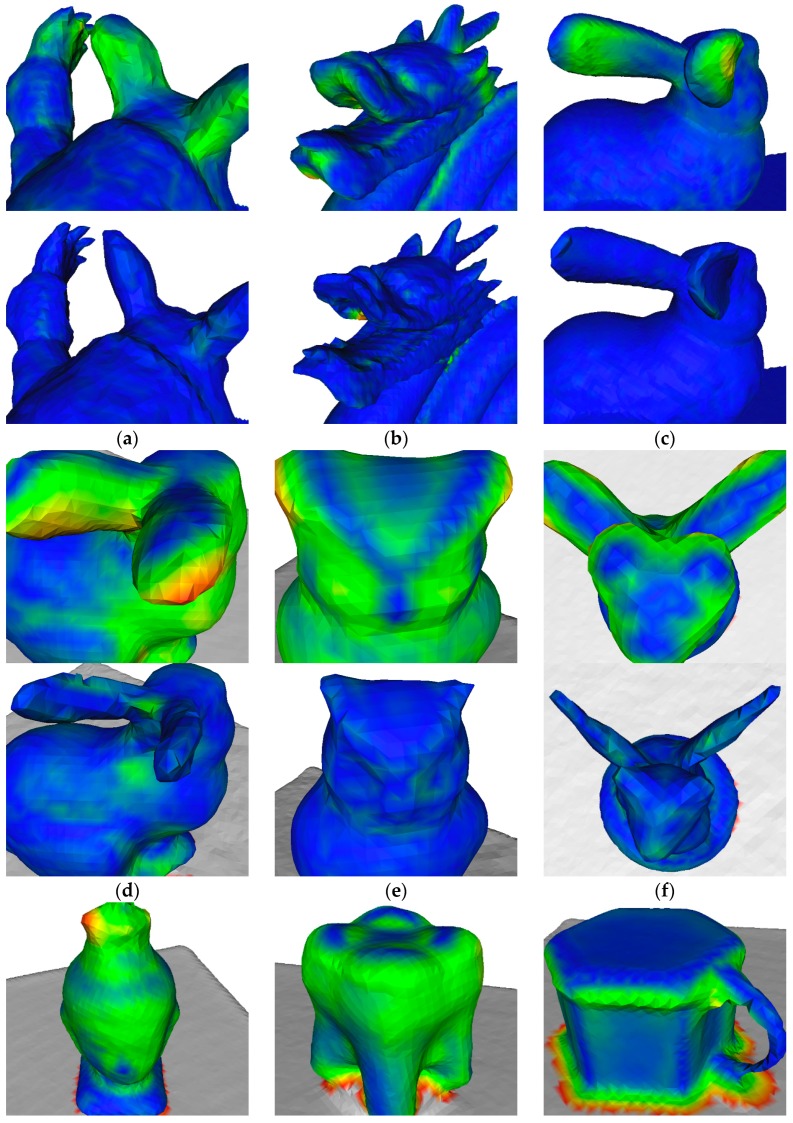

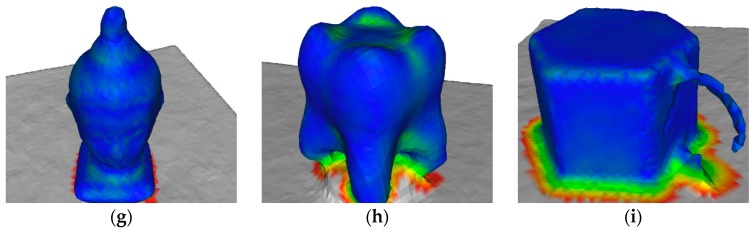
Close-up views of the reconstructions, colored with C2M distances. The synthetic data are (**a**) armadillo; (**b**) dragon; (**c**) bunny; and the real-world data are (**d**) lambunny; (**e**) owl; (**f**) wingedcat; (**g**) buddhahead; (**h**) tooth; and (**i**) mug. The odd rows are reconstructions of KinectFusion [1] as a comparison, and the even rows are of our approach.

We quantitatively evaluate the C2M errors for each algorithm with Mean and Std. statistics, as shown in Table 3 and Table 4. Our approach keeps the minimum values on both Mean and Std. in all experimental datasets, indicating its superiority in accuracy over the compared algorithms. Close-up views of the reconstructions are detailed in Figure 12, for further comparison between KinectFusion and our approach.

Additionally, we evaluate the reversed C2M errors, namely the distance from the point clouds of the GT models to the mesh of the surface reconstructions. ElasticFusion is excluded from this comparison, since it produces no surface meshes. Table 5 and Table 6 show the quantitative results of this evaluation. On average, the error of this metric is slightly larger than that of the normal C2M distance metric shown in Table 3 and Table 4, which results from the inabilities of the compared algorithms to accurately reconstruct extremely sharp surface geometries.

## 5. Discussion and Conclusions

We have presented a novel approach called CuFusion for real-time 3D scanning and accurate surface reconstruction using a Kinect-style depth camera. A man-made cuboid, the scale of which is accurately known, is used as a reference object for accurate camera localization without explicit loop closure detection, and a novel prediction-corrected TSDF fusion strategy is employed for reconstruction update. By solving the surface inflation problem introduced by the simple moving average fusion strategy, our approach preserves the surface details especially when scanning tiny objects or edge areas with high curvatures, resulting in high-fidelity surface reconstruction, which also improves the camera odometry accuracy in turn. We provide a dataset CU3D for the quantitative evaluation of our algorithm and have made our code open-source for scientific verification.

**Figure 13:**
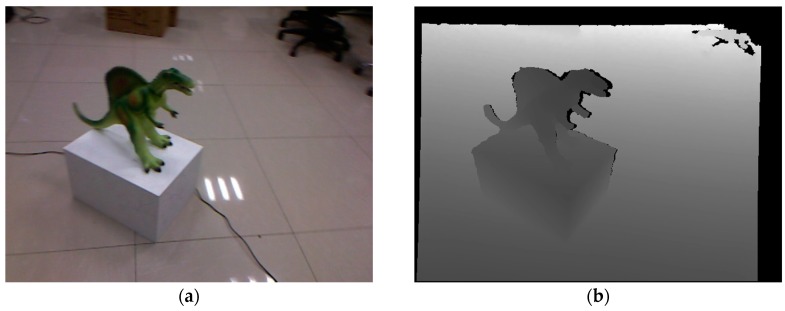

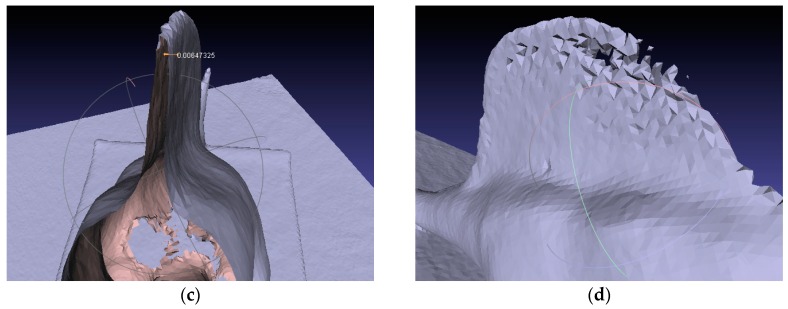
A failure case of our approach when scanning a Spinosaurus model with thin spines on its back. (**a**) Color (not used) and (**b**) depth image at time k=912 . Slightly faster camera motion around time *k* leads to mild motion blur, as can be seen from the color image; (**c**) A sectional view of part of the reconstruction before time *k*. Note how accurately our approach reconstructs the thin spines of the model; (**d**) A profile view of the reconstruction failure of the spine area at time *k*.

There are several limitations for future work to overcome. First, our modified dense volumetric representation needs 16 bytes per voxel—four times as much memory as KinectFusion at the same resolution—which limits our reconstruction to small-sized scenes. Second, to be capable of reconstructing high-curvature geometries, the camera should be moved as steadily as possible to reduce motion blur and uncertainty in depth measurements. Our algorithm trades off the robustness for reconstruction accuracy, which may fail in the presence of camera jitter or large motion. Figure 13 shows an example of the reconstruction failure result from depth motion blur artifact. Although no noticeable tracking drift happens, the reconstruction is delicate due to our prediction-corrected TSDF fusion strategy. Third, despite our efforts, the reconstructions are yet to be perfected due to sensor noise and the limitation of the volume resolution. As illustrated in Figure 12, engraved surfaces such as the armadillo shell, the facial expression of the owl, wingedcat and buddhahead are smoothed out—additionally, very thin geometries such as the owl’s ears and the mug’s handle are partly gone.

Our future work will focus on the memory efficiency of our modified volumetric representation, enabling higher volume resolution and a larger scale of reconstruction. The octree-based framework OctoMap [30] could be used for volume data compression. Another interesting challenge might be the surface smoothing problem, which we will focus on mitigating using the surface curvature consistency among the captured frames.

## Figures and Tables

**Table 1:** Camera intrinsic parameters used in our dataset, including the focal lengths $\left(f_{x},\;f_{y}\right)$ and the optical center $\left(c_{x},\;c_{y}\right)$ . Note that on real-world data the RGB and the depth camera share one intrinsic matrix $K_{r}$ since they are pre-aligned together.

Scenario	Intrinsic Matrix	$f_{x}$	$f_{y}$	$c_{x}$	$c_{y}$
synthetic	$K_{s}$ (RGB)	525.50	525.50	320.00	240.00
real-world	$K_{r}$ (RGB-D)	529.22	528.98	313.77	254.10

**Table 2:** Evaluation of the odometry accuracy with absolute trajectory error (ATE) RMSE metric in millimeters.

Synthetic Data	KinectFusion [1]	Zhou et al. [23]	ElasticFusion [20]	NICP [32]	Our Approach
armadillo	3.2	6.4	7.1	454.6	**1.5**
dragon	4.2	6.7	8.0	292.8	**1.7**
bunny	3.9	5.1	6.6	417.8	**1.3**

**Table 3:** Surface Reconstruction accuracy on our synthetic data, with C2M error metric ( Mean±Std. ) in millimeters.

Synthetic Data	KinectFusion [1]	Zhou et al. [23]	ElasticFusion [20]	Our Approach
armadillo	0.9±1.1	1.6±1.4	1.3±1.6	0.2±0.5
dragon	1.0±1.2	1.5±1.4	1.3±1.6	0.3±0.6
bunny	0.9±1.9	1.3±1.8	1.0±1.1	0.4±1.1

**Table 4:** Surface Reconstruction accuracy on our real-world data, with C2M error metric ( Mean±Std. ) in millimeters. Note that for real-world data, the evaluation is only performed on the 3D printed objectives but not the whole scene.

Real-World Data	KinectFusion [1]	Zhou et al. [23]	ElasticFusion [20]	Our Approach
lambunny	4.0±3.3	4.5±3.0	3.5±3.7	1.3±1.5
owl	4.4±3.1	4.9±2.9	5.1±4.4	1.1±1.3
wingedcat	5.0±3.3	5.2±3.1	3.2±3.1	1.5±1.8
buddhahead	4.8±3.3	5.3±3.0	4.5±3.7	1.0±0.8
tooth	4.4±1.7	4.8±1.8	3.9±3.6	1.2±1.0
mug	2.7±2.0	3.5±2.3	5.0±3.2	0.9±0.8

**Table 5:** Reversed C2M error in millimeters on our synthetic data.

Synthetic Data	KinectFusion [1]	Zhou et al. [23]	Our Approach
armadillo	1.7±1.5	2.0±1.5	0.5±0.6
dragon	1.8±2.1	2.0±2.2	0.9±2.0
bunny	1.4±2.3	1.7±2.3	0.9±2.3

**Table 6:** Reversed C2M error in millimeters on our real-world data.

Real-World Data	KinectFusion [1]	Zhou et al. [23]	Our Approach
lambunny	3.3±2.6	3.9±2.5	1.2±1.0
owl	4.0±2.1	4.8±2.1	1.5±1.6
wingedcat	7.6±5.9	8.0±6.0	4.3±5.0
buddhahead	4.7±1.8	5.6±1.6	1.1±0.8
tooth	4.1±1.6	4.6±1.7	1.0±1.0
mug	2.3±1.5	3.1±1.9	1.1±1.2
